# Initial experience with the Carina™ platform for gasless transaxillary robotic thyroidectomy in papillary thyroid carcinoma: an early feasibility report

**DOI:** 10.3389/fendo.2026.1804469

**Published:** 2026-07-03

**Authors:** Haiqing Sun, Xincheng Liu, Guibin Zheng, Chi Ma, Yang Liu, Jinyao Ning, Xicheng Song, Haitao Zheng

**Affiliations:** 1 Department of Thyroid Surgery, The Affiliated Yantai Yuhuangding Hospital of Qingdao University, Yantai, China; 2 Department of Otolaryngology Head and Neck Surgery, The Affiliated Yantai Yuhuangding, Yantai, China

**Keywords:** central lymph node dissection, modular robotic platform, papillary thyroid carcinoma, robotic thyroid surgery, transaxillary thyroidectomy

## Abstract

**Introduction:**

Rapid evolution in robot-assisted surgery have advanced the development of modular robotic platforms as innovative solutions. While several modular platforms have been reported in diverse surgical fields, their application in thyroidectomy remains unexplored. Here, we evaluated the feasibility and safety of the Carina™ modular robotic system for thyroid surgery.

**Methods:**

This preliminary prospective, single-arm study involved pre-clinical validation using porcine and cadaver models, followed by clinical implementation. Seven patients diagnosed with papillary thyroid cancer and scheduled for endoscopic thyroidectomy underwent gasless transaxillary robot-assisted thyroidectomy using the Carina™ platform. Surgical outcomes were analyzed to assess the feasibility and safety of the platform.

**Results:**

Seven patients underwent gasless transaxillary robot-assisted unilateral lobectomy and central neck dissection, without conversion to open or conventional endoscopic surgery. The mean system preparation time was 10.43 ± 3.99 (7–17) min, teleoperation time was 116.14 ± 24.20 (80–151) min, and operative time was 196.43 ± 29.40 (150–230) min. The physical and mental stress load scores of the surgeons were low. The average number of retrieved central lymph nodes was 6.14 ± 2.91 (2–11) with 1.00 ± 1.83 (0–5) metastatic nodes. Transient paresthesia was the most common complication in the subclavian skin region, but no severe complications were observed.

**Conclusion:**

This preliminary study suggested that the Carina™ system is a feasible and safe method for unilateral thyroidectomy with central lymph node dissection in highly selected low−risk thyroid surgery patients, with acceptable operative times and no severe complications in this initial clinical series.

## 1 Introduction

Most patients with papillary thyroid carcinoma (PTC) have a good prognosis. However, esthetics has become an important consideration in the surgical treatment of thyroid cancer. Postoperative neck scars seriously affect the quality of life of patients, and if possible, most patients prefer surgical approaches that avoid visible scars on the neck (1, 2). Both traditional and robot-assisted endoscopic thyroidectomies can achieve scar-free neck surgeries. Robot-assisted thyroid surgery, as an emerging technology, offers several technical advantages, such as a flexible mechanical arm with seven degrees of freedom, three-dimensional high-definition field of view, and tremor-filtering function. Since Kang et al. first applied robotic technology to thyroid surgery in 2007 (3), numerous studies have confirmed its feasibility and benefits (4–10). However, to our knowledge, all previous studies have focused on integrated surgical robot systems, such as the da Vinci system, and no studies to date have evaluated modular robot-assisted thyroid surgery.

**Figure 1:**
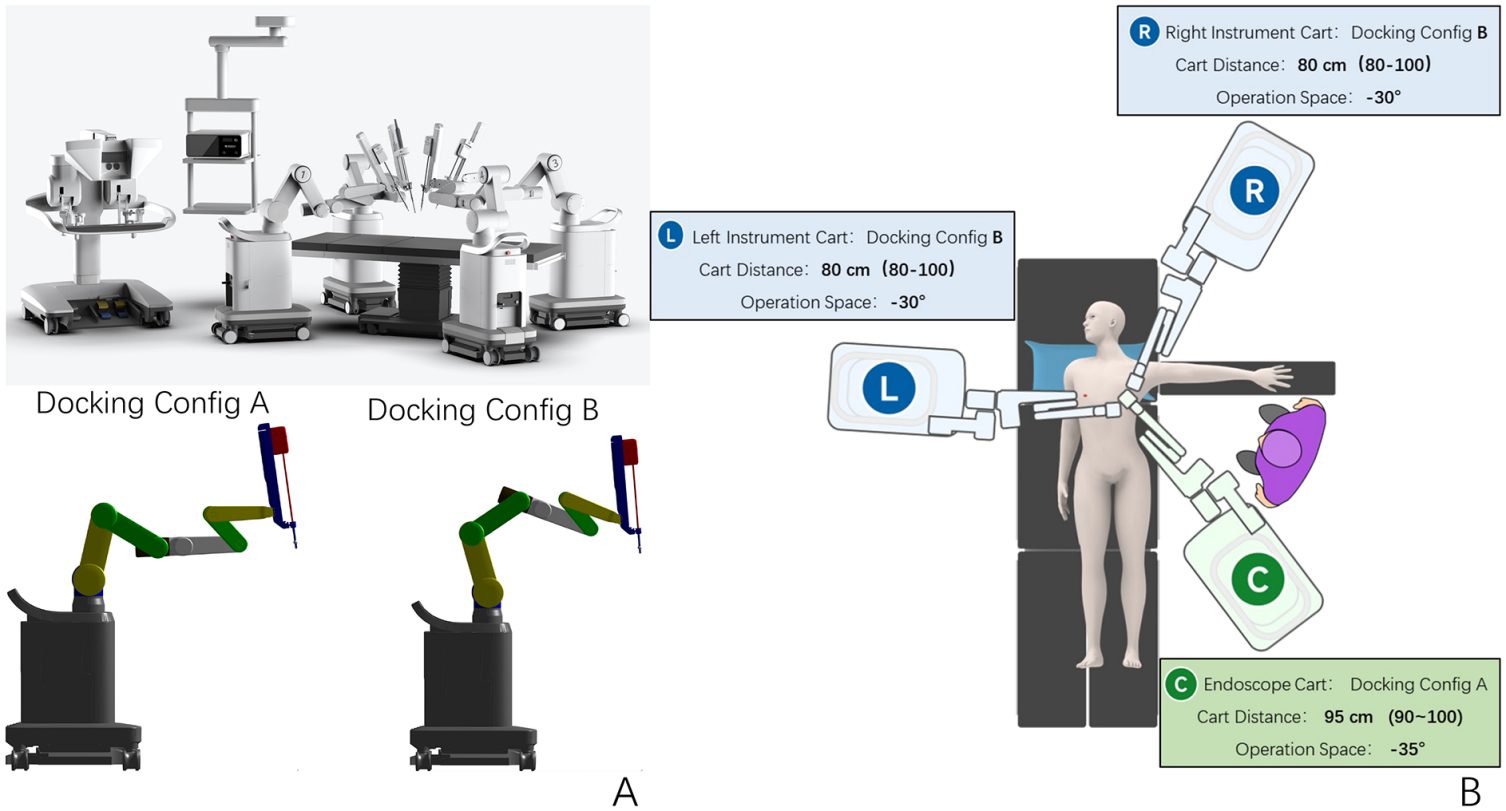
Carina™ Platform and patient cart positioning. **(A)**. Carina™ Platform and its docking configurations. **(B)**. Patient cart positioning.

The Carina™ Platform (Ronovo Surgical, Shanghai, China) is a newly introduced robotic system that received marketing approval from the National Medical Products Administration of China on March 6, 2025. The system was approved for use across four major specialties: general surgery, urology, gynecology, and thoracic surgery. Unlike the overall structural design of da Vinci, the Carina™ platform consists of an immersive surgeon console, four independent patient carts, and an integration hub (**Figure 1A**). Each robotic arm is installed on a separate patient cart, and once positioned, the carts are aligned through integration hub alignment (11, 12). This modular design allows greater flexibility in instrument layout during surgery (13), potentially offering advantages for the confined surgical workspace of thyroidectomy. However, clinical evidence for modular robotic systems in thyroid surgery study remains absent. To address this gap, we conducted a preliminary study to evaluate the Carina™ platform’s safety and technical feasibility for gasless transaxillary thyroidectomy with central lymph node dissection in selected patients with PTC.

## 2 Materials and methods

### 2.1 Patients and data collection

This preliminary prospective, single-arm study was approved by the Ethics Committee of Yantai Yuhuangding Hospital (Chinese ethics ID: 烟毓医伦理药/械审[2025]034号-01; English translation: Yantai Yuhuangding Hospital Ethics Review (Drug/Device) No. [2025] 034-01) and registered in the Chinese Clinical Trial Registry (www.chictr.org.cn; ChiCTR2500101419). Patients were enrolled between May 2025 and June 2025. The inclusion criteria were as follows: (a) age 18–80 years and (b) a diagnosis of PTC by fine-needle aspiration cytology with a maximum tumor diameter of ≤2 cm. The exclusion criteria were as follows: (a) body mass index (BMI) <18.5 or ≥30 kg/m²; (b) clinically significant comorbidities that precluded tolerance of endoscopic thyroid surgery; (c) extrathyroidal extension; (d) lateral neck lymph node metastasis or distant metastasis; or (e) history of surgery or radiotherapy in the neck or chest region; and (f) pregnancy or breastfeeding. All patients underwent preoperative neck ultrasound and enhanced computed tomography for tumor localization and evaluation of extrathyroidal invasion and lymph node metastasis.

We used multimedia tools to explain the novel system and clinical trials to patients to ensure they were fully aware of the possible benefits and risks. Patients were given the option to choose between the novel modular robot-assisted endoscopic surgery, traditional endoscopic surgery, and traditional open surgery, and provided written informed consent. All patients received the same standard pre- and postoperative care regardless of the surgical approach. Seven patients diagnosed with PTC, who underwent gasless transaxillary robot-assisted thyroid surgery, were included.

We collected data during and after surgery to assess feasibility and safety. The system preparation time was defined as the interval from connecting the first robotic arm to the trocar until the surgeon began controlling the instruments from the console. The teleoperation time was defined as the time spent by the surgeon operating the robotic arm on a control console. Operative time was defined as the interval from incision to closure. Redocking events, the number of recorded collisions, and subjective robotic arm interference score (1 = no interference, 5 = severe interference) were used to assess platform performance. The Local Experienced Discomfort (LED) and Subjective Mental Effort Questionnaire (SMEQ) visual analog scales were used to assess the physical and mental stress load of the surgeon (14). Postoperative pain intensity was evaluated using the visual analog scale on days 1, 4, and 30.

### 2.2 Carina™ platform

We utilized the Carina™ platform, which has been previously described in detail (11, 12). Three patient carts were employed: one laparoscopy cart equipped with a 30° laparoscope to provide surgical visualization, and two operation carts equipped with long fenestrated forceps, ultrasonic scalpels, or Maryland bipolar forceps for grasping, dissecting, and cutting. Before initiating the clinical series, we repeatedly verified and optimized the surgical procedure and patient cart placement using porcine and cadaver models.

### 2.3 Surgical procedure

After induction of general anesthesia, the patient was placed supine with the shoulders elevated, head rotated to the healthy side, and affected upper limb extended. A 5-cm incision was made along the first or second axillary fold on the affected side, without extending anteriorly beyond the mid-axillary line to ensure concealment. An additional 8-mm incision was made above the edge of the areola on the same side as the axillary incision.

The skin flaps were dissected along the pectoralis major muscle fascia under direct visualization with a headlamp, extending over the clavicle to the sternocleidomastoid (SCM). A specially designed automatic retractor was inserted through the main incision to elevate the skin flap over the sternal head of the SCM, facilitating identification of the gap between the sternal and clavicular heads.

The nurses and bedside surgeon jointly performed the docking procedures. Sterile drapes were installed over the robotic arms, and the docking and inclination angles of each patient cart were adjusted according to a preset configuration. Each patient cart was positioned beside the patient, and immobilization was applied. Subsequently, the handheld registration device was individually inserted into the patient-side carts to establish system connectivity. The trocars were docked to the robotic arms, and the camera trocar was adjusted into the workspace through the lower edge of the main incision, while the other trocars were adjusted into the workspace through the areola incision and the upper edge of the main incision. Finally, a 30° endoscope was attached to the camera arm, and the instruments were connected to the operational arm. The detailed position, distance, and angle of the patient cart are shown in **Figure 1B**.

**Figure 2:**
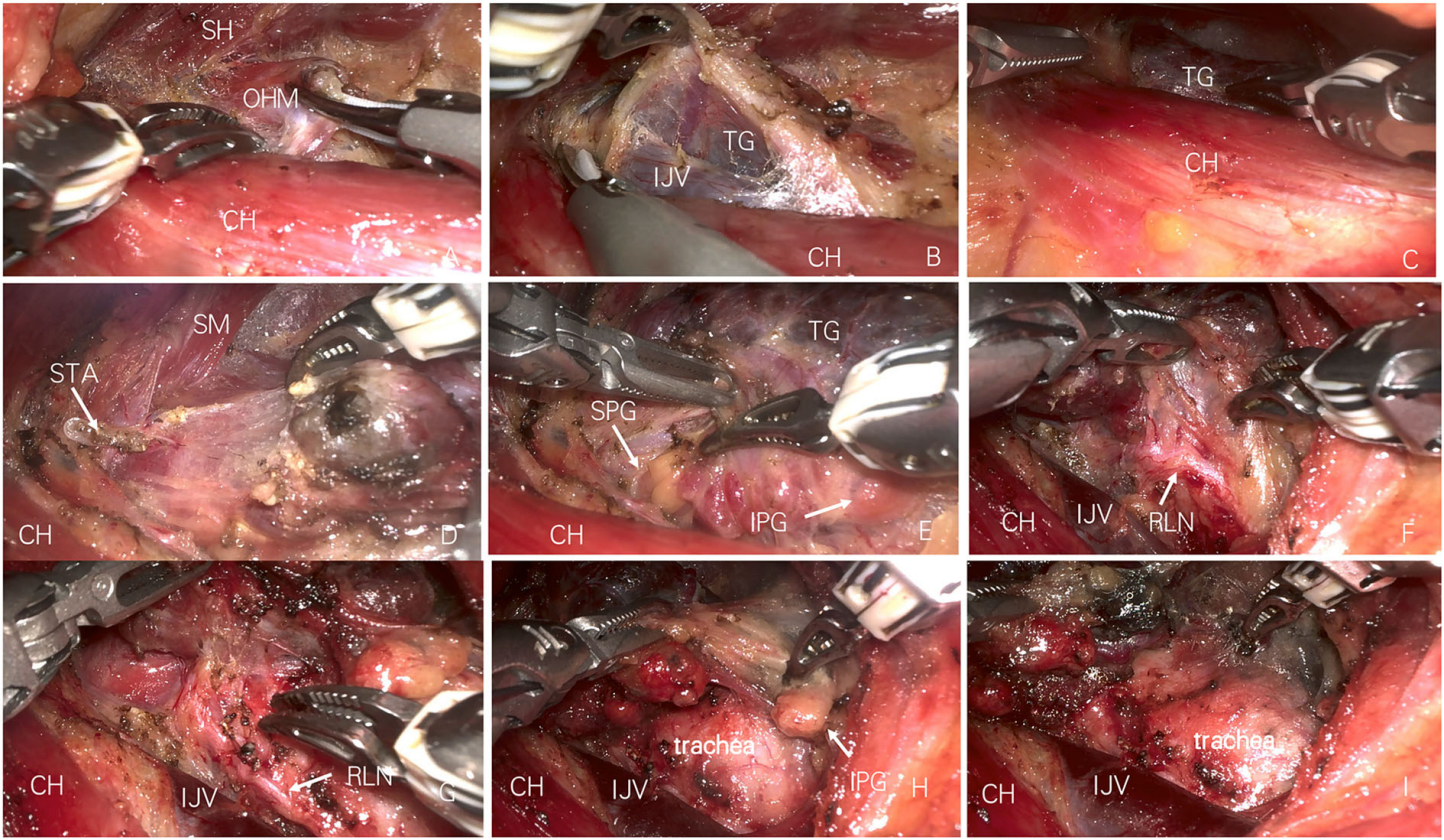
Surgical procedures. **(A)**. Omohyoid muscle was identified. **(B)**. Fascia between the lateral border of the strap muscle and the carotid sheath was dissected. **(C)**. Strap muscle was pulled forward using the automatic retractor. **(D)**. Superior thyroid vessels were sequentially transected. **(E)**. Superior and inferior parathyroid glands were identified. **(F)**. RLN was identified. **(G)**. Thyroid lobe was dissected from the trachea. **(H)**. Central fat-lymphatic tissue was cleared. **(I)**. Isthmus was dissected, and specimen was removed. SH, sternal head of SCM; CH, clavicular head of SCM; OHM, omohyoid muscle; TG, thyroid gland; IJV, internal jugular vein; SM, strap muscle; STA, superior thyroid artery; SPG, superior parathyroid gland; IPG, inferior parathyroid gland; RLN, recurrent laryngeal nerve.

The sternal and clavicular heads of the SCM were divided longitudinally with an ultrasonic scalpel until the omohyoid was identified (**Figure 2A**). The automatic retractor was adjusted to the rear of the sternal head of the SCM and pulled forward. The omohyoid was separated or severed, and the fascia between the lateral border of the strap muscle and the carotid sheath was dissected using an ultrasonic scalpel or Maryland bipolar forceps, exposing the thyroid gland (**Figure 2B**). A space was created between the strap muscle and thyroid gland, after which the automatic retractor was readjusted to pull the strap muscle forward (**Figure 2C**).

The upper pole of the thyroid was retracted inferiorly, and the branches of the superior thyroid vessels were identified and sequentially transected close to the thyroid capsule using Maryland bipolar forceps (**Figure 2D**). If the external branch of the superior laryngeal nerve crossed the superior thyroid pedicle below the upper border of the superior thyroid pole, it was identified during this step; otherwise, it was not specifically sought. The superior parathyroid gland was identified before transection of the posterior branch of the superior thyroid artery and was preserved *in situ* when an adequate blood supply was present. The inferior parathyroid gland was identified and gently mobilized laterally along the thymus or its vascular pedicle (**Figure 2E**). If either parathyroid gland was devascularised, autotransplantation was promptly performed to improve graft survival.

The thyroid gland was retracted medially using fenestrated bipolar forceps to expose the tracheoesophageal groove, and the recurrent laryngeal nerve (RLN) was identified and traced during meticulous dissection with Maryland bipolar forceps (**Figure 2F**). The thyroid lobe was then carefully dissected from the trachea (**Figure 2G**). Central fat and lymphatic tissue along the margin of the thymus or brachiocephalic artery were cleared (**Figure 2H**). The thyroid isthmus was divided (**Figure 2I**), and the thyroid lobe and central lymph nodes were removed *en bloc*. An aspiration drain was placed through the areolar incision, and the axillary incision was closed with a 5–0 absorbable subcuticular suture.

All procedures were performed by a single surgeon with 10 years of experience in endoscopic thyroid surgery but fewer than 20 prior robotic cases. Pathological assessment was conducted by two experienced pathologists. Following thyroidectomy, suppressive therapy with levothyroxine was initiated in all patients to achieve target thyroid-stimulating hormone levels in accordance with the 2015 American Thyroid Association guidelines. Patients were scheduled for outpatient follow-up at 1, 3, and 6 months postoperatively, with ultrasonography performed at 6 months to assess for recurrence.

### 2.4 Statistical analyses

All data were analyzed using SPSS software (version 26.0; IBM Corp., Armonk, NY, USA). Quantitative variables are described as means ± standard deviations, while categorical variable data are reported as numbers and percentages.

## 3 Results

### 3.1 Baseline characteristics

Between May 16 and July 1, 2025, seven patients diagnosed with PTC underwent gasless transaxillary robot−assisted unilateral lobectomy and central neck dissection using the Carina™ platform. All patients were women, with a mean age of 44 ± 11 years and a BMI of 24.90 ± 3.40 kg/m². Among them, four had Hashimoto’s thyroiditis, five had lesions on the left side, and two had lesions on the right side. The mean tumor size was 0.89 ± 0.32 (range, 0.5–1.3) cm. Five patients were diagnosed with classic PTC, one with infiltrative follicular variant PTC, and one with a combination of classic and infiltrative follicular variant PTC. (**Table 1**).

**Table 1:** Baseline characteristics.

Parameter	Mean ± SD or N	Range or percentage
Sex		
Female	7	100%
Male	0	0%
Age (years)	44 ± 11	(30–58)
BMI (kg/m^2^)	24.90 ± 3.40	(21.48–29.72)
Hashimoto’s thyroiditis		
Yes	4	57.15%
No	3	42.86%
Tumor location		
Left	5	71.43%
Right	2	28.57%
Tumor size (cm)	0.89 ± 0.32	(0.5–1.3)
Subtypes of PTC		
Classical	5	71.43%
Infiltrative follicular	1	14.29%
Classical and Infiltrative follicular	1	14.29%

BMI, body mass index.

### 3.2 Surgical outcomes

All seven patients successfully underwent gasless transaxillary robot−assisted unilateral lobectomy and central neck dissection using the Carina™ platform, with no conversions to conventional open or endoscopic surgery. The mean system preparation time was 10.43 ± 3.99 (7–17) min, teleoperation time was 116.14 ± 24.20 (80–151) min, and operative time was 196.43 ± 29.40 (150–230) min.

Redocking of the patient carts was required in three instances (the first two cases required adjustment of the camera arm angle and distance. In the fourth case, deviation in docking of the right instrument cart led to interference with the patient’s ipsilateral arm). In the first two cases, the number of collisions reached as high as 18 and 11, respectively, occurring mainly between the instrument and the camera or the automatic retractor. After optimization of the camera arm angle starting from the third case, the number of collisions decreased markedly, occurring only occasionally between the instrument and the automatic retractor. No instrument damage or patient injury resulted from the collisions. Subjective robotic arm interference scores were 4 and 3 for the first two cases, and 1–2 for the remaining five cases.

As described in the previous study by Grochola et al. (14). Immediately after surgery, LED was used to assess the surgeon’s upper body discomfort on a scale from 0 (no complaints at all) to 10 (extreme amount of complaints), and SMEQ was used to assess mental stress on a scale in which scores below 20 indicate little effort and scores above 110 indicate an exceptional amount of effort. The mean LED and SMEQ scores were 2.00 ± 3.06 (0–8) and 20.86 ± 11.26 (6–36), respectively, indicating low levels of physical and mental stress for the surgeons.

The mean postoperative drainage volume was 122.57 ± 65.79 (65–259) mL, and postoperative hospital stay was 3 ± 0.58 (2–4) days. Postoperative visual analog scale pain scores averaged 1.14 ± 1.07 (0–2) on day 1, increased to 1.71 ± 1.80 (0–4) on day 4, and decreased to 0.29 ± 0.76 (0–2) on day 30. The average number of metastatic central lymph nodes was 1.00 ± 1.83 (0–5), whereas the average number of retrieved central lymph nodes was 6.14 ± 2.91 (2–11) (**Table 2**).

**Table 2:** Surgical outcomes.

Parameter	Case 1	Case 2	Case 3	Case 4	Case 5	Case 6	Case 7	Mean ± SD or N	Range or percentage
System preparation time (min)	7	14	7	12	7	9	17	10.43 ± 3.99	(7–17)
Teleoperation time (min)	129	151	119	80	123	122	89	116.14 ± 24.20	(80–151)
Operative time (min)	200	230	200	150	225	205	165	196.43 ± 29.40	(150–230)
LED score	8	0	4	2	0	0	0	2.00 ± 3.06	(0–8)
SMEQ score	36	36	21	16	15	16	6	20.86 ± 11.26	(6–36)
Blood loss (mL)	10	10	10	10	10	5	10	9.29 ± 1.89	(5–10)
Redocking events	Yes	Yes	No	No	Yes	No	No	3	42.86%
Instrument collisions	18	11	6	3	4	3	2	6.71 ± 5.82	(2–18)
Subjective robotic arm interference score	3	4	2	1	2	2	1	2.14 ± 1.07	(1–4)
Postoperative drainage volume (mL)	259	108	85	147	113	65	81	122.57 ± 65.79	(65–259)
Postoperative hospital stay (days)	3	4	2	3	3	3	3	3 ± 0.58	(2–4)
VAS pain score									
Postoperative day 1	0	2	2	2	2	0	0	1.14 ± 1.07	(0–2)
Postoperative day 4	4	0	2	2	0	0	4	1.71 ± 1.80	(0–4)
Postoperative day 30	0	0	2	0	0	0	0	0.29 ± 0.76	(0–2)
Metastatic central lymph nodes	1	0	0	0	0	5	1	1.00 ± 1.83	(0–5)
Retrieved central lymph nodes	5	7	2	4	11	8	6	6.14 ± 2.91	(2–11)

VAS, visual analog score; PTC, papillary thyroid carcinoma; LED, local experienced discomfort; SMEQ, subjective mental effort questionnaire.

Transient paresthesia in the subclavian skin region was the most common complication, occurring in all seven patients and resolving spontaneously within 1–3 months. One patient developed a seroma 3 months postoperatively, which resolved after ultrasound-guided needle aspiration. No patients experienced complications, such as voice hoarseness, cough, aspiration, decreased vocal range, bleeding, infection, or chyle leak (**Table 3**).

**Table 3:** Postoperative complications.

Complications	N	Percentage
Transient paresthesia of the subclavian skin region	7	100
Postoperative bleeding	0	0
Hoarseness	0	0
Cough and aspiration	0	0
Decrease in vocal range	0	0
Seroma	1	14.29
Infection	0	0
Chyle leak	0	0

## 4 Discussion

The field of robot-assisted surgery is rapidly evolving. Although the da Vinci system dominates robotic surgery, more than 40 robotic surgical systems have been launched worldwide (15), typically at substantially lower cost to enhance competitiveness (16, 17). The modular robotic platform is an innovative design that enables rapid reconfiguration for different procedures, potentially reducing the learning curve for surgeons. Previous studies have reported the application of at least eight different modular platforms in various fields, such as colorectal, urology, hernia, and gynecology (11, 12, 15, 16, 18–23). The modular design reduces instrument-arm collisions and prevents patient compression by allowing real-time adjustments to arm positioning, angle, and cart distance. To the best of our knowledge, no prior reports have described the use of modular surgical robots for performing thyroidectomy, making this the first study to address this gap.

The most common surgical incision for gasless transaxillary robot-assisted thyroidectomy is the axillary anterior line incision (24–26), which optimizes the working space between the robotic arms and helps avoid intraoperative collisions. Following successful validation in animal and cadaver studies, we applied this incision technique to the first two human patients. However, the esthetic outcomes of the healed incision were unsatisfactory. The traditional gasless transaxillary endoscopic thyroidectomy commonly employs the axillary fold incision, which provides better cosmetic results (27–29). In clinical practice, we observed that adjusting the camera angle allowed the camera arm to maintain a certain distance from the inferior incision margin while preventing instrument collision, thereby enabling the use of the axillary fold incision to enhance cosmetic outcomes. This method was successfully implemented in five consecutive cases, demonstrating its clinical feasibility.

Instrument collisions and interference between robotic arms are the biggest obstacles to the use of the platform. In the first two cases, we performed redocking to adjust the camera arm angle and distance. In the fourth case, redocking was required because of interference between the right instrument cart and the patient’s arm. The same situation occurred during our initial cases of gasless transaxillary thyroidectomy using the da Vinci platform. A higher number of instrument collisions and higher subjective robotic arm interference scores were also observed in the first two cases. However, after optimizing the angle of the camera arm to position it in a different plane from the bilateral instrument arms, both outcomes improved significantly in the subsequent five cases. This preliminarily demonstrates the feasibility of the platform fir gasless transaxillary thyroidectomy for low-risk thyroid cancer while also revealing early challenges that decreased rapidly with operator experience.

The learning curve for robot-assisted gasless transaxillary lobectomy ranges from 20 to 66 cases (29–31). Our center has extensive experience with over 3,000 cases of endoscopic thyroidectomy via various approaches but limited prior experience in robotic thyroid surgery (less than 20 cases). All seven patients in this study successfully completed the robot-assisted procedure without requiring conversion to conventional laparoscopy or open surgery. System preparation, teleoperation, and total operative times were slightly longer than those reported in other literature (29–31), potentially reflecting the learning curve effect. All procedures were performed by a single surgeon with extensive experience in open and endoscopic thyroid surgery but no prior exposure to the Carina™ platform. The observed decline in surgeon physical and mental stress scores across the seven cases is consistent with a typical early learning curve associated with adoption of a new surgical platform and should primarily be interpreted as a familiarization effect with the new robotic platform. Due to the small sample size, no firm conclusion can be drawn regarding the number of cases needed to achieve stabilization of mental workload. Moreover, generalizability of these findings to other surgeons or settings may be limited. Although similar findings have been reported in other surgical applications of the Carina™ system, comparison of stress levels with those associated with conventional endoscopic surgery and other robotic platforms require further investigation.

In this study, the mean number of central lymph nodes retrieved was 6.14 ± 2.91, with 1.00 ± 1.83 metastatic central lymph nodes detected, which was slightly higher than that previously reported (29). Additionally, the mean number of retrieved central lymph nodes was marginally higher than that in our institution’s prior experience with conventional endoscopic transaxillary surgery (28, 32). This provides some support for the feasibility of performing central lymph node dissection using the platform. However, lymph node yield in the central compartment is influenced by multiple factors, including specimen handling, pathological examination practices, inflammatory background, and interindividual anatomical variation. The inclusion of 57.14% of patients with concomitant Hashimoto’s thyroiditis in this cohort may have contributed to interindividual variability. Further large-scale studies are needed to validate these findings and assess their clinical significance.

Postoperative pain was mild in all patients at days 1, 4, and 30, comparable to that in previous reports of da Vinci robotic transaxillary thyroid surgery (25). Multiple studies have demonstrated no significant differences in postoperative pain between robot-assisted endoscopic thyroid surgery and open thyroid surgery (33, 34). No severe complications were observed, confirming the safety of gasless transaxillary robot-assisted thyroidectomy using the Carina™ system. Patients reported transient paresthesia in the subclavian skin region, consistent with prior literature and attributed to flap dissection and infraclavicular cutaneous nerve injury (24). All cases resolved spontaneously by the 3-month follow-up.

The modular design of robotic platforms offers advantages in optimizing operating room space. In our study, we randomly selected an operating room adjacent to the storage room. The surgeon console was fixed in a corner of the operating room. The patient carts were stored in the storage room when not in use, and it took approximately 5–10 minutes to move them from the storage room to the operating room and position them around the patient when needed. Similarly, returning them to the storage room also took approximately 5–10 minutes. Unlike traditional integrated surgical systems, the modular design of robotic platforms, which require more operating room space owing to distributed patient-side carts, offers superior adaptability. Its compact individual carts can be installed in virtually any standard operating room and easily relocated between rooms (11). a feature similarly noted in the Hugo robotic system (35). Moreover, modular robotic platforms can be stored in warehouses or corridors when not in use, thus eliminating the need for permanent operating room space allocation. This storage flexibility is particularly valuable for hospitals with limited operating room capacity, as it allows robotic systems to be deployed only when needed, while freeing up critical space for other surgical equipment.

The current limitations of the Carina™ system were identified. Similar to most surgical robotic platforms, it lacks built-in vascular closure devices, such as Hem-o-lock clips or titanium clips, necessitating bedside assistance from surgeons to manually apply laparoscopic vascular clips for larger vessel occlusion (35, 36). Additionally, intraoperative blood and tissue fluids must be manually removed by the bedside team, along with specimen retrieval. The absence of indocyanine green fluorescence imaging functionality is another drawback that will be addressed in next-generation systems (12). Additionally, the standard trocar design lacks extended-length options, which may pose challenges for patients who are obese or taller; this limitation will be resolved in second-generation iterations (12).

This study has some limitations. The most important limitation is the highly selected low-risk patient cohort with a small sample size. Therefore, the favorable perioperative outcomes observed in this study are at least partly attributable to case selection rather than solely to the performance of the Carina™ platform. Second, our institution’s limited experience with robotic thyroid surgery precluded the inclusion of a da Vinci robotic system control group, and the relatively short follow-up duration may have underestimated long-term complications and outcomes. Third, only patients who underwent unilateral thyroidectomy were included, limiting a comprehensive assessment of postoperative parathyroid function. Forth, the learning curve associated with our early adoption of the Carina™ robotic system likely contributed to prolonged operative times compared to published benchmarks, potentially skewing the representation of the system’s efficiency in the hands of experienced surgeons.

In this initial series of seven patients, all procedures were successfully completed without conversion and with adequate oncological outcomes. The Carina™ platform demonstrated acceptable feasibility and safety in highly selected low−risk thyroid surgery patients, supporting the potential of modular robotic systems as cost−effective alternatives to integrated platforms, particularly for institutions with limited resources. However, these conclusions are strongly influenced by patient selection bias and the operator learning curve and therefore cannot be generalized without caution. Prospective comparative trials against integrated systems are needed to establish relative efficacy, cost−effectiveness, and long−term outcomes, along with evaluations of applicability to more complex cases and validation across multiple centers.

## Data Availability

The original contributions presented in the study are included in the article/supplementary material. Further inquiries can be directed to the corresponding authors.
